# Wind energy development in Norway: exploring the emotional landscape

**DOI:** 10.3389/fpsyg.2025.1386921

**Published:** 2025-02-14

**Authors:** Sigurd Hilmo Lundheim, Erica Löfström

**Affiliations:** ^1^Department of Sociology and Political Science, Norwegian University of Science and Technology, Trondheim, Norway; ^2^Department of Psychology, Norwegian University of Science and Technology, Trondheim, Norway

**Keywords:** wind energy, engagement, appraisal theory of emotion, opposition, emotions

## Abstract

**Introduction:**

In Norway, despite ambitious goals for a low-carbon society, the extensive extraction of fossil fuels persists, accompanied by widespread climate skepticism. Wind energy is proposed as a solution but faces resistance.

**Methods:**

This study examines the experiences of both developers and opponents of wind energy through qualitative interviews. Using appraisal theory, we classify emotional reactions, finding sadness and disgust as the most prominent negative emotions.

**Results and discussion:**

Additionally, fear and frustration were prevalent, reflecting tensions between wind energy and individual values. Emotional reactions vary widely and suggest that opposition to wind energy is multifaceted. Opponents exhibit stronger emotional responses, while developers, representing business interests, show less intense emotions. We identified 23 key triggers for these emotions, which often can be seen as disruptions caused by the development of wind energy. Engagement, comprising cognitive, affective, and behavioral elements, is essential to addressing these conflicts. Early engagement gives stakeholders the opportunity to influence the process, thereby reducing the conflict level. This highlights the need for earlier and more inclusive engagement processes to foster meaningful dialog and uphold democratic principles.

## 1 Introduction

Norway has an important role as an energy exporter. This is a result of large oil and gas reserves allowing Norway to export 87% of its energy in 2020 (IEA, 2022). The ongoing war in Ukraine has further cemented Norway’s position as an energy export country. The large extraction of fossil fuels stands in contrast with the ambitious goals to reduce greenhouse gas emissions by at least 50% before 2030 compared to the 1990 levels and to become a low-emission society by 2050 (not including the impact of exported oil and gas) (Klima og miljødepartementet, 2023).

A survey by the Norwegian fact-checking service, faktisk.no, found that one in four Norwegians believe that climate change is first and foremost natural (Varfjell, 2023). A study by YouGov found that that only 35% of Norwegians surveyed believe that the climate is changing, and that human activity is mainly responsible (Smith, 2019). In most Western countries, a much larger share of the population is convinced that human activity is the main force behind the current observed climate change. In the YouGov study, Norway tops the list of climate skepticism together with Saudi Arabia, whose economy also strongly relies on exporting fossil fuels. However, this should not be equated with outright climate denialism: according to the same study, most Norwegians believe the climate is changing, and human activity is partly responsible, together with other factors (Smith, 2019).

Wind energy is proposed as one way out of Norway’s fossil economic lock-in. One of the main advantages of wind energy, emphasized by its supporters, is that it provides climate-friendly energy (Rinaldo et al., 2024). In a country with many climate skeptics, this argument may garner little support, and the disadvantages of wind energy may lead to an escalation of wind energy opposition. The large availability of, and long traditions of, an alternative source of renewable and climate-friendly energy in Norway, hydroelectric power, may further increase the momentum against wind energy in Norway, even among those who are not climate skeptics. Norwegian electricity production is almost emission-free, with 88% coming from hydroelectric power plants and 10% from wind energy (Statista, 2023). Generating surplus energy for export may be a prominent economic motive, an argument whose punch is watered down by the involvement of foreign investors in wind energy consortia (Rinaldo et al., 2024). By 2023 69% of wind farms in Norway have foreign ownership (Pedersen, 2023).

Wind farms in Norway are typically built in relatively remote areas, but to keep construction costs down they are often located near existing infrastructure such as roads or powerlines. As a result, even though the areas of wind energy are sparsely populated, such developments will still impact residents. According to Statistics Norway (2024), a population total of only 89,018 inhabitants live in the ten counties with the most wind energy per square meter. Despite the relatively low number of people affected by wind energy development there has been a lot of opposition. In 2022 35% of the Norwegian population were against development of wind energy (Aasen et al., 2022). The two largest Facebook groups, *Nei til vindkraft Motvind Norge* and *Motvind*, had 118,000 and 55,000 members, respectively, in 2024.

Wind energy development is often a source of controversy. The controversy includes several topics such as sound or visual impacts, environmental concerns or issues of fairness, participation and trust (Rand and Hoen, 2017). This paper investigates the experiences of both developers and opponents involved in wind energy-related controversy, with a particular emphasis on the emotions these experiences evoke. Additionally, the study sheds light on the more general mechanism of status quo bias, which often emerges when national policies are enacted in local projects and face strong resistance. Such resistance is frequently attributed to NIMBYism, a term derived from the expression “Not in my backyard” meaning that one is negative toward the siting of something that might be unpleasant if it is close to where one lives, but not as long as the siting is far from home. This explanation have been challenged by researchers in the past (Devine-Wright, 2005).

Factors influencing acceptance and opposition to wind energy has been a topic of research internationally, e.g., (Langer et al., 2018; LaPatin et al., 2023). Our study examines different local developments in Norway and does not fathom more general international or national discussions on wind energy as such. However, by examining the qualitative experience of these conflicts, we aim to provide insight into how individuals with different roles relate to wind energy development. This approach may also uncover previously unknown factors and deepen our understanding of the key emotional triggers and arguments involved.

Our study addresses the following research questions:

(1) Which emotions are experienced by stakeholders in Norway’s wind energy controversy, and what might trigger these emotions?
(2) What are the key arguments in the controversy, and how do these arguments relate to the emotional triggers?

## 2 Theory

The theoretical framework for this paper is primarily grounded in the appraisal theory of emotions with a particular focus on climate-related emotions as defined by Pihkala (2022). Additionally, understanding engagement (Lorenzoni et al., 2007) is important, in both interpreting the emotional sides of the wind energy controversy and facilitating democratic processes in wind power development. We also consider disruption as a possible trigger for some of the emotional reactions to wind energy. Finally, we use previous literature on *ownership*, as research has found that different forms of ownership structures can influence attitudes toward wind energy particularly by amplifying positive attitudes and suppressing negative attitudes (Warren and McFadyen, 2010).

### 2.1 Appraisal theory of emotion

**Figure 1:**
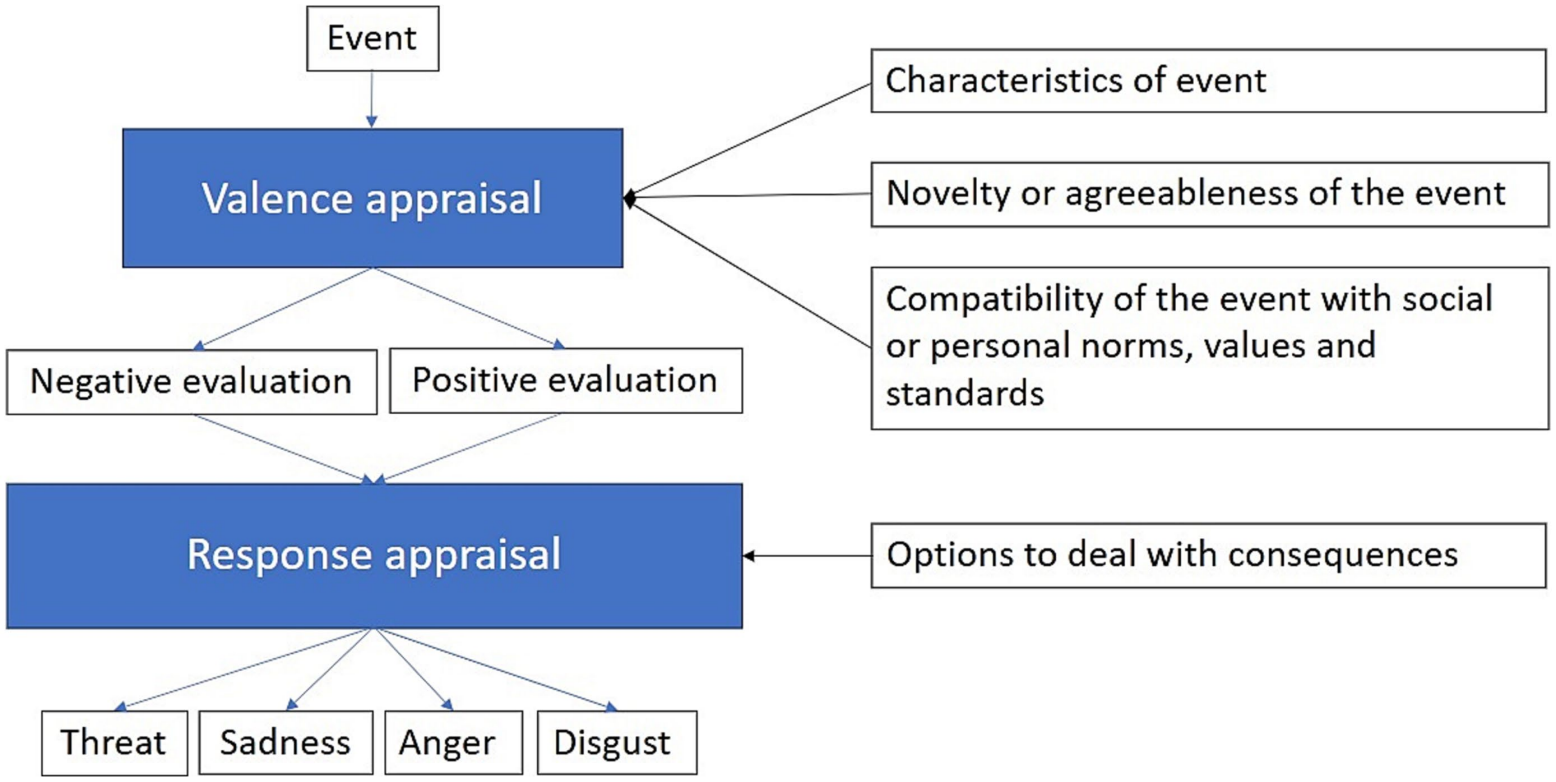
How an event is appraised based on its characteristics and compatibility with personal norms and values. This appraisal determines the valence (positive or negative) and leads to corresponding emotional reactions. In this case, the focus is on negative emotions such as threat, sadness, anger, and disgust.

Emotional reactions to events are a key part of human life and can also be an important motivator (Izard, 2007). How humans react emotionally to an event can be predicted from their cognitive appraisal. However, when discussing emotions, it is necessary to reach a common understanding of emotions. This is easier said than done, there is not a universally agreed upon definition of emotions (Ortony, 2022). For the purpose of this paper, we define emotions as “feelings that are directed at someone or something” (Hume, 2012). We use appraisal theory (Scherer, 1999) to get a better understanding of how these emotions occur. Appraisal theory posits that emotions are elicited in two stages. The first stage evaluates whether the event is positive or negative for individual well-being. The second stage evaluates if the individual can deal with the consequences of the appraised event (Scherer, 1999). According to appraisal theory, there are four main sets of criteria that people use to evaluate events: (1) The intrinsic characteristics of the event, which are focused on the novelty or agreeableness of the event, (2) how important the event is for a person’s needs or goals, (3) the individual’s ability to cope or influence the consequences of the event and (4) the compatibility of the event with social or personal norms, values and standards (Scherer, 1999). Figure 1 illustrates our understanding of appraisal theory of emotion and provides a parsimonious representation.

According to Scherer (1999), one aspect of the event’s novelty is the suddenness, which could trigger emotions such as anger and fear – both of which are reflected in our data. Appraisal theory also accounts for the diversity of emotional reactions to wind energy. Appraisal theory addresses both positive and negative emotional reactions; however, our study focuses specifically on negative emotional reactions. Therefore, the figures presented illustrate only the negative emotional reactions that we identified, categorized according to the climate taxonomy presented by Pihkala (2022). Appraisal theory has guided several other papers concerning the emotional responses to renewable energy technology (Perlaviciute et al., 2018; Vespa et al., 2022).

Nerb et al. (2001) found that the appraisal of the magnitude of environmental impact, in this case an oil spill, influences the intensity of emotions like anger and sadness. Additionally, Nerb and Spada (2001) suggest that anger is the more likely emotion if the event has an apparent entity to blame. In contrast, if there is no obvious entity to blame, sadness is the more probable response, though both emotions may still coexist.

### 2.2 Climate emotions

To inform our analyses we use the taxonomy of climate emotions by Pihkala (2022), which provides a nuanced framework of emotions experienced as a response to climate-related events. Given the complex variety of emotions present in our data, we opted for Pihkala’s detailed taxonomy over simpler models, such as Ekman (1992) basic emotions theory, to better capture the depth of participants’ emotional responses. Several negative emotions found in this paper result from perceived negative changes in the local environment. In this case there are many participants with a biospheric value orientation (de Groot and Steg, 2007). This makes climate emotions an appropriate theory as much of its foundation is in the emotional reactions caused by climate change events. Further the emotional taxonomy presented by Pihkala (2022) also includes emotions tied to morality such as disgust or resentment.

The need for a nuanced emotional taxonomy was further supported by the concept of emotional granularity – the idea that people vary in how precisely they express emotions, with some using precise specific terms and others relying on more general expressions of pleasure or displeasure (Barrett, 2004). For this reason, we used Pihkala’s detailed taxonomy to categorize climate emotions. The selected emotions are listed in Table 1.

**Table 1:** Climate emotions relevant for this study.

Emotions and mental states	Variations of climate emotion
Threat-related emotions	Fear, worry, anxiety, feeling overwhelmed (in mild or moderate amounts)
Sadness-related emotions	Sadness, solastalgia
Anger-related emotions	Anger, frustration
Disgust-related emotions	Disgust, resentment

### 2.3 Engagement

#### 2.3.1 Stakeholder engagement

Lorenzoni et al. (2007) define engagement as an individual’s *state*, comprising three elements: cognitive, affective, and behavioral. This emanates from the idea that people must care about the issue and be motivated to take action (Lorenzoni et al., 2007). By including these aspects, engagement reaches beyond just the idea that people need to possess knowledge to be engaged. Even though local engagement and conflicts may sometimes be perceived as annoying by developers and politicians, one should acknowledge that conflicts and acts of resistance among citizens are legitimate parts of a functioning democracy.

Lack of stakeholder engagement, particularly in the early stages of a construction project, can be highly problematic. When stakeholders are not involved early on, they have less opportunity to develop counterarguments against the project. Early engagement also provides room for adaptations and negotiations. Moreover, supporters are unlikely to get engaged if they feel the construction as inevitable, which limits the development of psychological ownership. Previous research highlight stakeholder involvement early in the planning process and recommends prioritizing openness, dialog and active engagement (Durham et al., 2014; Solman et al., 2021).

#### 2.3.2 Barriers and enablers for engagement

In Europe today, there is a general understanding of climate change as a serious problem (European commission, 2023). However, knowledge alone is not sufficient to motivate individuals to engage in climate action. It is important to preface that engagement can manifest in different areas. In the context of wind energy development much of the discussion on engagement is related to climate policy and energy infrastructure. Several barriers hinder participation in climate change-related action. Lorenzoni et al. (2007) presents two main categories of barriers: (1) Individual barriers, such as lack of knowledge, uncertainty, skepticism and distrust in information sources; and (2) social barriers, often related to external forces, such as lack of political action, lack of industry initiative, and prevailing social norms and expectations. Lorenzoni et al. (2007) emphasizes that “Local environmental issues are not only more visible to the individual but present more opportunities for effective individual action than climate change.” This observation is relatable to the recent wind energy controversies, as resistance is often seen as combatting local environmental issues. Such conflicts can highlight a tension between global climate mitigation efforts and local environmental conservation. The relative proximity of wind turbines to a local community makes them appear much closer in physical and temporal space compared to the more abstract concept of climate change. Moreover, Klöckner and Löfström (2022) note that climate change scenarios and cost-related actions to deal with climate change are often perceived as uncertain, further complicating individuals’ willingness to engage. Naturally, preservation of the local environment is more manageable and works as a “framing” of the problem to a geographical area. In addition, the timeframe of a local wind power development may be perceived as more manageable than that of the more elusive general climate change process. These two “limiting” factors may well explain the relatively high level of residents’ engagement regarding local wind power developments.

### 2.4 Disruptive communication

The development and implementation of renewable energy technologies has the potential to interrupt everyday life. The transition from internal combustion engines to electric motors in cars demands changes of habits related to charging, removal of oil burners for heating is expensive and the construction of wind turbines can lead to noise and disturbance of scenic views. This disruption often provokes an emotional response. Disruption can also be used strategically and is then called disruptive communication. In short, confronting established assumptions and disrupting people’s everyday lives provokes emotional engagement. The end goal of disruptive communication is to create a situation that allows for radical change to take place (Klöckner and Löfström, 2022).

Previous literature has identified wind energy development as a source of disruption, i.e., it can disrupt the place attachment (Devine-Wright and Howes, 2010). In Norway, resistance to wind energy development has been both active and highly visible in the media. Particularly, the indigenous population has been active in protests, even winning lawsuits against the Norwegian state regarding wind power developments that threaten their traditional way of life (Fjellheim, 2023). Kim and Chung (2019) found that the establishment of a wind farm can make memories of earlier place disruption more salient. This finding is particularly relevant for Norway, where the indigenous population has had their traditional land expropriated for other purposes on several occasions. Consequently, the development of wind farms represents yet another disruption of their sense of place attachment.

### 2.5 Ownership

The ownership of wind energy has been found to influence the acceptability of wind energy; see Lundheim et al. (2022) for a literature review. Community ownership, in all its forms, has been shown to effectively enhance acceptance. One frequently cited example of successful community ownership is the wind farm at the Danish Island of Samsø. A key factor for the resident’s acceptance is the possibility for residents to become co-owners of the local wind turbines (Löfström, 2015). Local ownership, and the absence of foreign or outsider investors, have contributed to the fact that the locals on Samsø have changed their perception of the wind turbines in the local environment. Comparative studies further support the link between community ownership scheme and higher acceptance rates (Musall and Kuik, 2011; Walker and Baxter, 2017). However, Leer et al. (2020) found that community ownership and a property value loss compensation scheme did not compensate for the non-economic factors of wind energy development. In the following section we discuss engagement, and we believe that for any local or cooperative ownership to occur, stakeholders must be able to engage with the project.

## 3 Methods

The sampling for this paper was done by employing purposive sampling (Etikan et al., 2016). More specifically, we employed critical case sampling as this work is exploratory, and we cannot draw wide generalizations.

The study is based on qualitative, in-depth interviews (Rutledge and Hogg, 2020) with two different stakeholder groups in Norway: developers of wind power projects and opponents. In total, nine interviews were conducted, two with developers and seven with opponents of wind energy. According to Rutledge and Hogg (2020), in-depth interviews are an appropriate method to gain insight into the participants experiences and feelings regarding a specific topic. The opponents represent community organizers of resistance to wind energy, selected from different online discussion groups. This group of people represents important figures in the Norwegian wind energy resistance as they are significant contributors to the online activity of the opposition. The biggest opposition group in Norway has 118,000 members (in 2024), which is a significant number of members considering that the country has a population of 5.46 million. We chose this sample as we believe that these Facebook groups could provide valuable insights into why people are in opposition to wind energy. This is also supported by Borch et al. (2020). Despite the open nature of the discussion groups, we chose not to name them to keep the participants anonymous. The second group of participants were found by contacting those companies in Norway that were engaged in planning and building of wind farms at the time of data collection. Due to a lack of response from several companies, the decision was made to move forward with those that provided timely replies. Two representatives of companies were willing to participate. Again, we do not name the company or any identifying factors to guard their anonymity. Our study is not focused on a specific wind energy project since the stakeholders are involved in different projects. The inclusion of developers allows us to get a glimpse into the emotional reactions of both opponents and developers of wind energy.

We interviewed nine persons individually, seven opponents and two developers. Each interviewee was assigned a unique ID number (interview 1–9) for anonymously referencing their quotes, and we added “(Res)” or “(Dev)” in addition to making clear which sample the interviewee was from.

The participants represent different genders and age groups. However, we did not ask specifically about this, nor did we ask about education. We do this to adhere to the data minimization principle (Goldsteen et al., 2022). Another reason is that the number of opposition groups in Norway is limited, and we interview moderators of these groups. This could make it too easy to identify our participants, thus breaching researcher participant confidentiality. We acknowledge, however, that these factors may be necessary for other questions related to wind energy or other renewable technology.

The interviews were rich in data covering a wide range of topics. Data collection was planned to be done in person. However, they were collected on the tail end of the COVID lockdown in Norway. This was a very uncertain time therefore the interviews ended up being done online. Despite this limitation, the resulting data is extraordinarily rich. Much of this is because our participants are involved to a considerable degree in the controversy. This involvement leads to our participants having a lot of experience as stakeholders in their respective wind energy conflicts.

### 3.1 The interview guides

We used two interview guides to gather our data (Appendix 1). One guide was created to interview opponents of wind energy projects, and the other guide was created to interview the developers of wind energy projects. Both interview guides are informed by previous work on acceptance of wind energy research and are closely related to each other but nevertheless contain individual components. The interview guide to those opposing wind energy covers in broad terms the following topics: Initial awareness of wind energy development, attitudes toward wind energy, perceived ownership related to wind energy, community benefits, environment impact, effects on nature, and personal experiences of the conflict. The developer questionnaire differs in that it asks less about the impact of wind energy and more about the perception of opposition. All interview guides were tested aforehand to ensure that these are non-ambiguous and would allow for different people to understand the questions in a similar way.

### 3.2 Data analysis

Four criteria are widely used to appraise the trustworthiness of qualitative research: credibility, dependability, confirmability and transferability (Denzin and Lincoln, 1996; Guba and Lincoln, 1994). The authors have carefully addressed each of these criteria to achieve a high level or trustworthiness. Central to this effort is transparency in data collection and analysis. Given the limited population size and the even smaller number of individuals directly affected by local wind energy developments, much of the debate and resistance has taken place online in different social media groups. We used these platforms to recruit participants, aiming to talk to as many individuals as possible who are either directly affected or actively involved in the discussion. Although our number of participants is modest, we have successfully identified representatives who offer insight that reflects the ongoing debate. All of the respondents representing opponents were moderators of their respective Facebook groups or they were referred to as key figures by other members of these groups. This paper aims to explore the diverse experiences of both developers and opponents of wind energy in relation to the controversy surrounding it, with a particular emphasize on emotions evoked by these experiences.

The analysis was done individually by the authors, followed by a comparison and discussion of findings. To facilitate the analysis, the authors employed a qualitative data management tool known as NVivo, which allows researchers to keep a clear overview of the codes and themes (Dhakal, 2022). The process was both reflexive and iterative (Pyett, 2003). To analyze our data, we used thematic analysis (Clarke et al., 2015), informed by the literature on appraisal theory (Scherer, 1999) and climate emotions (Pihkala, 2022). This method enabled us to identify the stakeholders’ emotional reactions to wind energy development.

Thematic analysis was chosen for its flexibility, allowing us to adopt an organic approach to the data, where themes naturally emerge during the analysis process. It should be noted that the emotional classifications are grounded in our impressions of both the content and delivery of what was said during the interviews. This approach gives us a clearer understanding of the underlying emotions reflected in the quotes. We selected quotes that represent the interviewees perspectives in a fair manner and did not pull quotes out of context.

The original data is in Norwegian, and all quotes were translated using DeepL (DeepL, 2023) and human translators.

### 3.3 Limitations

This paper is based on interviews with nine persons engaged in different wind energy projects in Norway, and thus we cannot generalize the findings to a broader context. Worth mentioning is that it was challenging to find representatives from the developers’ side who were willing to be interviewed. Further, when interviewing the developers of wind energy, it was clear that they felt more constrained during the interviews compared to the individuals who were opposed to wind energy.

It should also be mentioned that this paper focuses solely on onshore wind energy, however, offshore wind energy is gaining recognition as an important major step in wind energy development (Shen et al., 2024).

The data was collected online due to COVID pandemic restrictions. Not being able to visit the people that took part in the interviews meant that it was not possible to observe sites of protests or significant areas for the stakeholders. This makes us somewhat removed from the local development that is often the source of controversy.

## 4 Results and discussion

This paper explores several different emotional reactions to wind energy, both among wind energy opposers and those who are actual developers. Based on these emotions, we have identified overarching themes that trigger these emotional reactions among our participants. We identified and classified these emotional reactions inspired by previously defined categories (Pihkala, 2022, see Table 1 for details). Our analysis indicates that the emotional reactions to wind energy are quite similar to the emotional taxonomy identified by Pihkala (2022). However, we hold off on calling these categories universal as many of the countries studied so far – ours included – are so called WEIRD (Western, Educated, Industrialized, Rich and Democratic (Henrich et al., 2010).

### 4.1 The respondent’s emotional reactions

The respondents emotional reactions are structured in the same manner as the climate emotions identified by Pihkala (2022). We present respondents’ quotes and discuss the emotional reactions and their underlying causes. While certain emotions, such as worry and anxiety, overlap and are sometimes interconnected in other contexts (Sweeny and Dooley, 2017), we have chosen to structure the results and discussion according to distinct emotional themes. We begin by examining threat-related emotions, followed by those associated with sadness, as shown in Table 1. Each quote is labeled with a unique identifier (Q1, Q2, Q3…Q27).

#### 4.1.1 Threat related emotions

The following section addresses emotions that are related to the assessment of threat, including emotions such as fear, worry and anxiety, as well as helplessness and feeling overwhelmed.

##### 4.1.1.1 Fear

Several participants express fear when faced with wind energy, either explicitly or through responses that matches the appraisal structure of fear.

Quotes from the interviews:

(Q1) *“My house, for example, is not included in the noise calculation at all, but I’m one of the people who gets it the worst. Yes, it goes into the house, I’ve been having migraines since this has been set… and I haven’t had them before. I had a nosebleed here on Sunday, never had it before. Heart palpitations, [I] don’t sleep well because you go to bed with such a high pulse that your heart is beating, and it’s like that… just like that…”* (Res 4)

(Q2) *“What I’m afraid of is noise, the direct impact of noise, and some people have started to report that they suffer from more headaches, migraines, sleep less well and [it is generally] louder… [some] find this now constant noise very disturbing.”* (Res 5)

(Q3) *“They could have told the truth. […] These mills are full of poison… it’s composite plastic, right, that sticks together and there’s no way to get rid of it now. It’s a tragedy. The ones that are taken down now are either shoveled down or sent to African countries and paid for. It’s a huge environmental problem with them, in just a short time.”* (Res 6)

Also, the developers acknowledge fear among opposition members:

(Q4) *“They argue that house prices are falling in the areas so that the housing will not be worth anything, in other words, a series of scare tactics that … and some people become genuinely afraid of it and think ‘this is dangerous,’ ‘we just have to stop this,’ ‘this is… we could die here,’ ‘this is dangerous not only for humans, but also for… animals, especially birds.’”* (Developer 1)

###### 4.1.1.1.1 The triggers

The quotes show that triggers of fear can be health related, as well as environmentally related. Health concerns were common among opponents, with health fears mentioned in six of nine interviews. For example, Q1 and 2 expressed fears about potential health effects, including migraines, sleep disturbances, heart palpitations, and noise-related annoyance.

Environmental concern also emerged as a significant trigger. Participants raised issues such as biodiversity loss and the toxic materials in wind turbines (pollution). For instance, Q3 highlighted the environmental risks posed by composite plastic waste from turbines, framing it as an ecological tragedy. Fears also extended to the environmental impact of the turbines’ production and disposal at the end of the lifespan.

Developers are aware of the fear experienced by some opposition members and believe that certain individuals of the opposition deliberately amplify these fears by painting doomsday scenarios (Q4). These scenarios often involve claims about declining property values and health risks to humans and animals. However, developers frame these fears as something caused by propaganda. This is elaborated further in the discussion of “frustration.”

###### 4.1.1.1.2 Discussion of fear

Songsore and Buzzelli (2014) and Rubin et al. (2014) found that health-related concerns were a significant factor in public resistance to wind energy. However, the causal relationship between negative health effect and wind energy is not well established. A literature review by Knopper and Ollson (2011) attributed much of the health effects from wind turbines to annoyance. A more recent review found much of the same (Karasmanaki, 2022). Despite the lack of a well-established causal relationship, it is essential from an acceptability perspective to take these claims seriously. Fears of health issues can be a powerful motivator for resistance against wind energy. Karasmanaki (2022) suggests that objective information about wind turbines could be a way to mitigate these fears, however, motivated reasoning will most likely limit the impact of objective information (Druckman and Bolsen, 2011). Dismissing such complaints when the person experiences these issues could lead to greater frustration or anger (Jeffery et al., 2013).

Studying media coverage on wind turbines in Ontario, (Deignan et al., 2013) found that 94% of the articles focused on health effects, amplifying dread about potential impacts. Reports of health issues linked to wind energy, while likely not caused by the turbines themselves (Karasmanaki, 2022; Knopper and Ollson, 2011), are perceived as real. These experiences, and their narratives, often shared through news and social media provides anecdotal evidence raising opposition or at least instill skepticism.

Much of the fear is related to environmental impacts, a topic that has been extensively addressed in research (Adeyeye et al., 2020; Warren et al., 2005). Wind turbines are hard to recycle, and the different materials require different processes for recycling (Jani et al., 2022). Turbine blades, in particular, are notably difficult to recycle (Andersen et al., 2014). However, efforts to address these challenges are actively worked on (Jensen and Skelton, 2018). Environmental organizations, such as the Norwegian “Naturvernforbundet,” fear that the development would negatively impact biodiversity (Naturvernforbundet, n.d.), however in our analysis biodiversity loss is more strongly connected to worry.

##### 4.1.1.2 Worry

Another emotion identified in the data is worry, which like fear, reflects a feeling of stress (Levy and Guttman, 1985). Levy and Guttman (1985) highlight an asymmetric relationship between worry and fear: While fear is always accompanied by worry, worry can occur independently of fear.

Quotes from the interviews:

(Q5)*“They think it’s perfectly fine to build it in the middle of the bird migration where the radar measurements show that it’s densest, that’s where they’ll have turbines, they see that as unproblematic, and then they haven’t done any surveys in the ground or in the peat bogs. So they don’t know how deep it is, they don’t know what the soil is like, it’s an island so there hasn’t been ice there during the last ice age, so a lot of the rock has deeply weathered […] but generally speaking, you have to dig up and then fill it with concrete to make it stable. And that’s not something that has been predicted to happen, but it’s assumed that these will be small interventions.”* (7 Res)

(Q6) *“Oh, I knew about it, and it was probably in about 2014 while they were working on the 2013 construction. I don’t actually remember. It may have been as early as 2009, …, because that’s when the other landowners were told about the other [project] […] it wasn’t a big concern then, it was just ‘green energy’ and ‘saving the world’ and climate this and climate that … I knew that it might make some noise or take some birds, so I wasn’t quite sure if it was such a good idea, but it wasn’t something I cared much about then.”* (8 Res)

###### 4.1.1.2.1 The triggers

The primary triggers for these quotes stem from concerns about environmental impacts, safety issues associated with wind turbine construction and lack of early concern.

The first quote focuses on technical and ecological shortcomings, such as geological instability and impacts on wildlife. The second quote highlights lack of early concern. Interestingly, worry was not triggered instantly. Q6 describes a lack of worry during the initial planning stages, indicating that early indifference was later replaced by growing doubts as more information or consequences became apparent. This shift was influenced by the framing of the project as “green energy,” tied to goals like, “saving the world” and addressing climate change, which initially muted potential worries.

###### 4.1.1.2.2 Discussion of worry

The delayed worry expressed in Q6 is in accordance with the concept that wind energy resistance follows an inverse U-shaped curve (Devine-Wright, 2005), particularly evident in the first half of the curve. The respondent initially displayed little interest in wind energy and was not actively engaged in the development process. According to Lorenzoni et al. (2007), this lack of engagement may result from the absence of perceived disruption or changes in the local environment, making the impact of wind energy on the environment less salient. Furthermore, this initial lack of worry might lead to negative emotions in the future, as participants might feel regret for not acting when they had the opportunity.

While renewable energy is generally associated with less worry compared to fossil fuel-based energy (Burger, 2012), Q5 illustrates that renewable energy can still raise significant environmental concerns. The participant frames wind energy development as worrisome in this context, believing that it results in substantial changes to the local environment.

Burger (2012) investigated worry associated with different means of energy production, finding that fossil, chemical, and nuclear energy evoked the highest levels of concern, while renewable energy generated the least. Despite apparent similarities in negative emotions, previous research by Levy and Guttman (1985) highlights distinctions between them. Worry can affect stakeholders in wind energy development for different reasons. For instance, Enevoldsen and Sovacool (2016) noted that developers often worry about project delays, whereas politicians tend to worry about public outcry. Additionally, the perceived environmental impacts of wind turbines can trigger significant worry. Klain et al. (2018) found that concerns about harm to birds and marine mammals outweighed worries about economic costs. This aligns with findings from our interviews, which indicate that worry can stem from a variety of factors.

The visibility of wind turbines may act as a constant reminder of these worries. Experiencing ongoing worry could, in turn, trigger more severe emotional reactions such as anxiety (National Institute of Mental Health, 2022).

##### 4.1.1.3 Anxiety

Like fear and worry, fear and anxiety are closely related emotions. The distinction between them is not as clear-cut as previously believed, rather, it is complex and nuanced (Daniel-Watanabe and Fletcher, 2022). However, a distinction between fear and anxiety is helpful in guiding research. One of the clearest differences is that fear typically arises in response to a real and immediate threat, while anxiety is a response to a potential future threat (Catherall, 2003).

Quotes from the interviews:

(Q7) *“It doesn’t help to scare us with what’s happening now with temperatures rising and all sorts of things now, if we don’t have nature, we have nothing to live on.”* (6 Res)

(Q8) *“So the countries that have more wind power, they also have a much more expensive power supply, because they have to double down. They have to have double the supply, they have to have double the back-up. In Norway we have hydropower, but otherwise it’s gas power that acts as back up.”* (8 Res)

(Q9) “*What helps us in our opposition to wind power is this outcry against high electricity prices now. Not an electricity crisis, but an electricity price crisis. So, an alliance has formed here now, which is very interesting.”* (6 Res)

(Q10) *“You get a fairly significant reduction in property value, and some may not sell at all, which means that the property value is virtually zero.”* (8 Res)

(Q11) *“It’s terribly unfair, it’s an unfair taxation of some people who live right next to a wind turbine that they don’t want, and then they lose half the value of their house and home, and they don’t get anything back for that in Norway.”* (8 Res)

###### 4.1.1.3.1 The triggers

The triggers for these quotes reveal underlying concerns about environmental priorities (loss of local nature), economic implications, economic unfairness, and property impacts tied to wind energy development.

In the first quote (Q7), the trigger lies in the tension between global climate messaging and the local destruction of natural environments. The respondent expresses frustration with climate alarmism that fails to consider the immediate importance of nature conservation, emphasizing that without nature, broader environmental goals are meaningless. With the term “nature” the respondent probably refers to intact nature and ecosystems that are not influenced by human land use.

Secondly, the assumed increase in energy costs serves as a significant trigger. The economic inefficiency of wind power, particularly the expense of maintaining redundant energy systems to compensate for its intermittency, is highlighted in Q8. This stands in contrast to Norway’s reliance on hydropower, which provides a more stable and reliable energy source, supplemented by gas backup. Further, respondent 6 (Q9) view the current high energy prices as beneficial for the resistance to wind energy.

The triggers in the last two quotes (10 and 11) are related to the financial and emotional impacts of wind turbines on nearby residents. These include drastic reductions in property value and the inability to sell homes. The picture painted is quite bleak, describing a situation in which people will either be forced to sell at a much lower price or be stuck with their property because of the construction of wind turbines in the area. The emotional impacts include the perceived injustice of bearing the burdens of wind energy development without adequate compensation. Together, these quotes underscore how localized economic and social inequalities fuel opposition to wind projects.

###### 4.1.1.3.2 Discussion of anxiety

Anxiety related to the climate is often referred to as eco-anxiety, which is an emotional and mental reaction to environmental conditions and awareness of ecological issues (Pihkala, 2018).

Quote Q7 acknowledges the effects of climate change but suggests that preserving local nature is a higher priority than addressing global climate. This phenomenon, known as a green-on-green conflict (Warren et al., 2005) highlights competing environmental priorities. Both sides of the conflict use arguments rooted in environmentalism: one side emphasizes the clean energy benefits of wind turbines, while the other side points to the impact on the landscape. The quote is also interesting as it shows how an opponent might weigh the urgency of these two issues. While they acknowledge the severity of climate change, the thought of losing their local nature is perceived as a greater threat.

There is also evidence that anxiety is linked to concerns about energy supply (Karasmanaki and Tsantopoulos, 2019). This is particularly relevant given that one of the disadvantages of wind energy is its unreliability, which could lead to anxiety about consistent energy supply (Thayer and Freeman, 1987). However, research exploring the relationship between anxiety, eco-anxiety, and wind energy—or renewable energy more broadly—remains limited, highlighting a significant gap in the existing knowledge base.

One concern raised is that countries with larger investments in wind energy tend to face higher electricity costs (Q8). It is important to consider this statement within the context of when the data was collected, as energy prices were quite high in Norway at that time. Moreover, the concern over rising energy costs is described as something that is beneficial for wind energy resistance (Q9). This is particularly noteworthy as it suggests that at least some opponents of wind energy do not perceive wind energy development as a reliable way to lower electricity prices, either in Norway or abroad, as noted by participant 8 (Q8). Additionally, it shows the opposition’s ability to leverage a widespread source of anxiety to advance their cause effectively.

Losing property value was mentioned by multiple participants in the interviews (for instance Q10 and 11), and is also a topic frequently discussed in literature. Hoen et al. (2011) found that neither the view of wind turbines, nor the distance to them, had any statistically significant effect on the sales prices of the properties examined. However, other researchers have found that wind turbines do have a negative effect on property value (Heintzelman and Tuttle, 2012). A more nuanced view is presented by von Detten et al. (2023) assessing the effects of wind turbines on standard land values. They found that land values decreased more in areas with lower population density compared to those with higher population density. Wind energy in Norway is often built in areas that are sparsely populated, therefore a possible reduction in property values near wind farms in Norway is a relevant concern.

##### 4.1.1.4 Overwhelmed

Some of the participants described feeling helpless in the face of the different wind energy projects. Feeling overwhelmed and helpless are often seen concurrent in research (O’Neill and Nicholson-Cole, 2009; O’Neill et al., 2013). Therefore, we are classifying “feeling helpless” same as “feeling overwhelmed.”

Quotes from the interviews:

(Q12) *“And then it’s also quite overwhelming because when you start to see that you’re going to oppose a development that the state wants and that a large company wants, where do you start, but it wasn’t … it wasn’t informed where … as it is on … when they build a road, you get a letter that the appeal body is this and that, it didn’t exist for this wind power plant.”* (5 Res)

(Q13) *“Yes, it’s probably… there are two things. Firstly, it’s probably the fact that the local population feels completely run over.”* (6 Res)

(Q14) *“No, the main reason is what we were talking about, namely that it’s the loss of something dear to them. And then there’s the loss of being heard. The feeling and experience of being run over by something you never asked for.”* (2 Res)

(Q15) *“I thought for a while that it was possible to help prevent the massive destruction. Well, it’s changed in the sense that it’s become far more serious than when I started. And we haven’t yet progressed beyond the first round, the first phase. Because now both the EU and Støre [the Norwegian Prime Minister] are planning a new investment in wind power, and that will come next year.”* (2 Res)

(Q16) *“I actually sent a message to the Mayor saying, ‘You know what, now I’ve realized why we don’t hear anything from these people (other opponents of wind energy).’ They’re actually broken. They’re psychologically broken from being in this fight and having it shoved down their throats at the same time.”* (4 Res)

###### 4.1.1.4.1 The triggers

Lack of information and influence, fighting against powerful government bodies and corporations are significant triggers of feeling overwhelmed.

Respondent 5 (Q12) points at lack of information as a trigger to feeling overwhelmed. The respondent is lacking information on the plans for the project like the ones that are mandatory for other types of physical development like roads. At the time of the interview wind power development was not regulated by the Norwegian Plan and Building Act. This has now changed, and the planning process is more similar to the procedures for other physical development (Kommunal- og distriktsdepartementet, 2024).

Quote 15 reflects on how the respondent’s attitude toward opposing wind energy has changed. Initially, they believed it was possible to stop the destruction of local nature caused by the construction of wind farms. However, as they over time have not been able to influence the process, it has become increasingly clear that stopping it may be beyond their control. The feeling of helplessness can be very negative as individuals can become “destroyed” by their opposition to wind energy (Q13 and 16). The combination of opposing wind energy and not being able to influence the development of wind energy in a significant way is particularly destructive (Q14 and 16).

Many participants highlighted the perceived disparity in power between local opponents and large entities, such as government bodies and corporations. A perceived imbalance between developers and wind energy opponents, who feel like they are fighting both the state and large corporations, is expressed (Q12). This sense of battling overwhelming odds can create significant emotional distress. Further, the opponents also give the impression that they were left out of the process as they were not properly informed. This could exasperate the feeling of being overwhelmed as changes seem to suddenly happen. Several examples show people who feel like they have been “run over” by the wind energy developers (Q13 and 14).

##### 4.1.1.4.2 Discussion of feeling overwhelmed

In many cases the locals’ ability to oppose wind energy development is limited. At best, they may only succeed in delaying it (Aitken, 2008). This could lead to the opposition feeling overwhelmed by the other parties in the conflict. Ransan-Cooper et al. (2020) looked at emotional responses to siting solar batteries in Australia and found that feeling overwhelmed by the complexity of the process leads to less positive emotions. It is likely that similar emotional responses occur in the context of wind energy development.

Previous research has identified the planning process as an important factor of acceptance of wind energy (Lundheim et al., 2022; Ottinger et al., 2014; Walker and Baxter, 2017). Wind energy developers in the USA, along with environmental activists, felt that the planning process was too long and frustrating (York et al., 2016). Frustration from opponents was linked with more negative attitudes toward wind energy (Devine-Wright and Howes, 2010). The quotes do in our opinion, lend credence to this claim. By feeling overwhelmed they also give the impression that they feel left out of the process. This is supported by quote 14, pointing out that this is something that the residents never asked for. Participants felt excluded from the planning process. This lack of transparency and consultation contributed to their sense of being “run over.”

Russell and Firestone (2021) indicates that feeling helplessness is common among residents in areas undergoing wind energy development. In contrast, people who move to these areas after the construction is completed do experience less helplessness and show a more positive attitude. This difference could be a result of newcomers not having experienced the same disruption associated with constructions. In interviews, two quotes (Q15 and 16) give the impression that being in opposition to wind energy and not achieving their desired outcomes is an emotionally taxing experience – one that newcomers do share.

#### 4.1.2 Sadness-related emotions

This section addresses two sadness related emotions labeled sadness and solastalgia. In the present study, sadness-related emotions were experienced both by developers and opponents.

##### 4.1.2.1 Sadness

Sadness related to wind energy development in Norway is a recognized phenomenon (Normann, 2021). In an analysis of Twitter posts, Corbett and Savarimuthu (2022) found sadness to be the dominant negative emotion in discussions on sustainable energy.

Quotes from the interviews:

(Q17) *“Perhaps the first reaction was that they are taking our local nature, which is in a way the most important quality in these outskirts, as it probably is for all outskirts, it’s nature, nature experiences. And that was destroyed, not just on top of the mountain, but in a large surrounding area.”* (5 Res)

(Q18) *“You meet people who actually want to hurt you. […] It’s okay that they don’t wish me well, […] you meet people who want to hurt us because they think that we do something to bother them.”* (1 Dev)

###### 4.1.2.1.1 The triggers

One trigger that could be seen in quote 17 is the degradation or loss of local nature and possibilities for outdoor life. This loss causes emotional distress and feeling of sadness. These quotes emphasize how vital nature is for the community’s identity and well-being.

Based on the quotes from one of the developers (Q18), the willingness of wind energy opponents to consider inflicting physical harm can be identified as a trigger for sadness for developers. This trigger evokes the experience of being targeted by others, or of a possible radicalization of the opponents, which leads to feelings of sadness.

###### 4.1.2.1.2 Discussion of sadness

Quote 17 highlights the loss experienced by rural residents due to wind energy development, emphasizing their close connection to nature, which is integral to Norwegian cultural heritage. They describe the development as an assault on the values of local communities, framing it as a “robbery” (Q19).

Sadness arising from wind energy development could be understandable, as it diminishes the natural beauty and emotional bonds residents have with their surroundings. This sadness is intensified by the emotional connections to one’s environment (Casakin et al., 2021).

The sadness felt my developers reflects a confrontation with individuals who deliberately seek to harm or disrupt, causing distress and fear. The sadness expressed seems to be a result of the animosity that is targeted toward the developers. The animosity could be tied to the health danger perceived by the opponents. Additionally, it also illustrates how different the stakeholders’ perceptions are. Sadness, just as other emotional responses, has a strong influence on the support of climate mitigation projects, with emotional impacts being strongest for those living closer to such projects (Hart et al., 2015).

##### 4.1.2.2 Solastalgia

Solastalgia is a concept that refers to the distress people feel when environmental change affects their home while they remain connected to it (Albrecht et al., 2007).

Quote from the interview:

(Q19) “*And it is in the classic nature conservation or in the daily use of our natural qualities that we, the population, find ourselves. That’s where we live our lives, and that’s where we have our values, through outdoor life and other nature experiences. And that’s where the great robbery happens, which makes people sick and desperate.*” (2 Res)

###### 4.1.2.2.1 The triggers

Similar to Q17, Q19 expresses a sense of loss, or disruption of place attachment, feeling that wind energy development has harmed their future and their local environment.

###### 4.1.2.2.2 Discussion of solastalgia

The language further underscores the strength of their attachment to the area, describing that the developers have “destroyed” their local nature, which is considered the most important quality of their community. This sentiment reflects a profound sense of place attachment. The emotion evoked by this disruption of place attachment could be seen as a form of solastalgia in line with the findings of Phillips and Murphy (2021). Previous studies have also linked construction of wind turbines to solastalgia due to their impact on local environments (Mueller and Brooks, 2020). In general, our data also shows a strong connection to nature among participants. In other studies, emotional reactions to visual disruption due to landscape alteration has been classified as fear (Pasqualetti, 2011), in the present study solastalgia was found to be the most appropriate classification.

#### 4.1.3 Anger related emotions

Several emotions can be related to anger. In our analysis we have classified both anger and frustration as anger-related emotions.

##### 4.1.3.1 Anger

Quotes from the interviews:

(Q20) *“I think people have been so distraught that it is perceived as so unfair. It’s all been done in such a wrong way that we’ve had no chance and then you get quite angry, of course, and distraught so you express that, but also the supporters or I know even those who have made agreements, that regret it and have tried to get out, but it’s not possible.”* (5 Res)

(Q21) *“But of course, harsh words have been used, that’s true, but I feel that they’ve been driven from pillar to post. In the end… you get terribly angry, and you use words and phrases that you wouldn’t use under normal circumstances.”* (6 Res)

(Q22) *“One of the first things I did was to react to Germany’s policy, actually. Because I sat down that summer and read the Norway-Germany strategy…and I remember posting a comment about this. “Yes, those who don’t believe in this” or something like that, “they should read this.” And then I got a response like “Oh my God, you can’t bring the Germany strategy into this.” I don’t think that person would have said that today [laughs] two years later.”* (4 Res)

###### 4.1.3.1.1 The triggers

The most prominent triggers in the cited quotes are a perception of unfairness, lack of influence, defeat and distrust in politicians.

The first quote (Q20) highlights a sense of powerlessness and mistreatment, as participants perceive that decisions were made unfairly, without their input or the opportunity to influence the outcome. This trigger is closely tied to feelings of injustice and regret, with some participants expressing that they feel trapped in agreements they are unable to escape.

Frustration and being pushed into difficult situations provoke extreme emotional responses, as seen in Q21. The trigger here is the feeling of defeat, which causes individuals to use harsh language and act in ways they would not under normal circumstances. It reflects how anger can distort behavior and communication. The trigger in the final quote (Q22) is rooted in distrust in politicians. The participant’s reaction shows how deeply political policies can stir emotions, provoke strong response that led to public debates and conflicts in communication. This trigger is tied to broader political dissatisfaction and the way public discourse evolves over time.

###### 4.1.3.1.2 Discussion of anger

The anger felt by those impacted by wind energy industry has significantly influenced the ongoing debate, both in person and online. Quote 21 justifies the anger felt by people opposed to wind energy: Firstly, people have been driven from one perceived defeat to another, left them feeling concerned. Secondly, these perceived defeats have made them so angry that they use language that they would not have used in other circumstances. In their perspective, this anger could be seen as a form of righteous anger resulting from something evil or harmful.

To further illustrate that the tone of the debate can become quite confrontational, the development of wind energy in Norway is compared to the Norway-Germany strategy (Q22), probably referring to the plan made by Nazi Germany during World War 2 to export electric energy from Norway (Thrane and Johansen, 2022). We believe that the reason for this comparison is that at least six of the companies that invest in wind energy in Norway are German and the largest of them are involved in ten wind farms (Pedersen, 2023). This anger, and the comparison to the invasion plan from World War 2, could be because the participant feels like there is an invasion of Norway. According to the opposition, wind energy development damages local nature while it directs the economic benefits to owners far removed from the local community. Despite acknowledging that people who are opposed to wind energy experience anger toward the development, comparing said development to the nazi invasion is an extreme comparison.

Olson-Hazboun (2018) found that in fossil fuel-based communities in the US, a shift to renewable energy was a source of anger. Previous research shows that anger is often tied to process related grievances. Conversely, positive feelings such as pride were correlated with trust, project awareness and perceived benefits (Russell and Firestone, 2022). Additionally, anger is not only reserved for those who oppose wind energy. Opposition can also be a trigger of anger to those who do not oppose wind energy, as they believe that wind energy is part of the solution to climate change (Dunlap, 2018). The wide array of different effects wind energy has on both people and nature could result in negative attitudes and these attitudes could trigger negative emotions such as anger (D’Souza and Yiridoe, 2014). This is a factor that makes wind energy a complex topic as both opposition and advocation has the potential to lead to anger.

##### 4.1.3.2 Frustration

The present study shows that frustration is an emotion that is shared by both developers and opponents of wind energy development.

Quotes from the interviews:

(Q23) “*So, if we’ve tried to present facts about production in relation to area and how many households and so on, but they may not want much of that, they’d rather have a few tears [laughs], pictures and that we have nothing sensible to say, just emotional people*.” (5 Res)

(Q24) “*So, it has simply felt unfair, it’s no secret. You hear from key politicians that ‘We support you and this is good, but we can’t say anything until after the election,’ and then after the election they don’t say anything then […] it was a unanimous Parliament that adopted the renewable energy initiative in Norway and the wind power initiative, so…. it’s not something we made up*.” (Dev 3)

(Q25) “*I think it’s a very irresponsible way of making arguments and educating the public. Because you create… create a few scares in people, and some people become genuinely afraid of it.*” (Dev 1)

###### 4.1.3.2.1 The triggers

The triggers for the opponent’s frustration (Q23) are related to how the wind energy opposition are depicted by the media. The participant expresses frustration with how their efforts to present logical, fact-based arguments are disregarded in favor of emotional appeals. This creates a sense of being devalued or dismissed as “just emotional people” rather than being taken seriously for their rational perspectives.

Developer’s quote (Q24) reflects feelings of frustration, triggered by lack of political support. Politicians who promote wind energy often avoid addressing the controversies, leaving developers to manage the opposition alone. The lack of action after elections and the perception of being misled contribute to a sense of unfairness and disillusionment. Developer 1 (Q25) also express frustration caused by the misleading or fear-based messaging used irresponsibly, causing genuine fear and anxiety among people. This fearmongering is thus a trigger for the developer’s frustration.

###### 4.1.3.2.2 Discussion of frustration

Opponents of the wind energy development projects feel frustration due to the feeling of not being heard, as the media prioritize emotional reactions rather than factual information. Gross (2007) found that communities affected by wind energy development often experience frustration due to the perceived lack of impartial and scientific information. The media’s focus on emotional appeals instead of factual arguments contributes to the belief that their concerns are dismissed. This is further compounded by the absence of objective, evidence-based information. These frustrations are consistent with Gross’s findings, which highlight how communities often feel excluded and that their concerns are not taken seriously when they are not presented as impartial or scientifically supported.

Developers experience a lot of frustration tied to the political process surrounding development of wind energy. These feelings are tied to the unfairness experienced by them when development of wind energy is a political decision. From the point of wind energy developers, the politicians have pushed for development, but they do not want to engage the opposition and find it easier to leave the developers to deal with the controversy.

The authors have previously discussed the health concerns some people of the opposition have when concerning the effects of wind energy. The developers (Q25), believes that the statements related to health are deliberately made to create a climate of fear surrounding wind energy, see also section Fear. These statements are naturally a source of frustration for developers as they motivate the opposition to take action to stop wind energy development.

#### 4.1.4 Disgust-related emotions

Disgust and resentment were expressed during the interviews. We have classified these as disgust-related emotions.

##### 4.1.4.1 Disgust

Previous research has identified expressions of disgust in communication and interviews with wind energy opponents (Reusswig et al., 2016). Disgust has also been associated with opposition against policies aimed to mitigate climate change (Smith and Leiserowitz, 2014).

Quote from the interviews:

(Q26) *“Pacific islands that are supposed to disappear under the sea and then it turns out that they have grown. They are rising, but still, you send billions to them to support them with something or other. There are probably some corrupt top politicians down there who benefit from it.”* (8 Res)

###### 4.1.4.1.1 The triggers

The quote suggests that doubts about the legitimacy of climate measures can trigger disgust. Possibly this disgust is caused by the feeling of being fooled by the authorities and distrust in politicians believing that there are other motives behind the policy than mitigation of climate change. The quote expresses actions meant to mitigate climate change and the results of these policies. The participant was quite disgusted by the idea that Norwegian funds are being used to enrich “corrupt top politicians.” This is in line with the findings from Smith and Leiserowitz (2014). For people who are skeptical about climate change as is evident from quote 26 regarding the pacific islands it can be inferred that they may also question some of the anticipated impacts of climate change.

##### 4.1.5.1 Resentment

Resentment can be a result of either real or perceived injustice. Further, resentment can also be derived from inequity and in the case of wind energy development much of the anger is directed at politicians and other people perceived to be in a higher social stratum (Banning, 2006).

Quote from the interviews:

(Q27) “*And then it’s difficult like now with the climate crisis, there should have been many who dug into it, too, that it’s also mostly just fraud and misery, at least it’s miserable measures that don’t help anything. And that billions should be wasted on all sorts of idiotic projects. Politicians should have been impeached, so… someone should have addressed that and written about it, but it’s a bit like if you open your mouth and say something against the climate then. I feel it’s gotten a bit better there, too, maybe recently, […] there’s so much censorship, if it’s not proper censorship then it’s self-imposed censorship and then it’s the media houses… what they want to print.*” (8 Res)

###### 4.1.5.1.1 The triggers

Several different triggers for resentment could be found in the quote: The respondent expresses significant resentment with what they see as ineffective or misguided responses to the climate crisis, describing the measures as “miserable” and “idiotic projects” that do not provide meaningful solutions. Furthermore, a wasteful use of resources and lack of political accountability could be identified as triggers. The call for politicians to be “impeached” reflects a deep dissatisfaction with leadership and a perceived lack of accountability for poor decision-making. Finally, the resentment is also triggered by censorship and the media. The participant highlights frustration with censorship—both imposed and self-imposed—and media bias. They feel that open discussion about alternative perspectives, particularly those critical of climate policies, is stifled, contributing to a sense of being silenced or marginalized.

###### 4.1.5.1.2 Discussion of resentment

The resentment expressed in Q27 toward politicians is not uncommon: Other participants in the interviews have implied that politicians do not care about the environment and that this is only a play to give money to companies or individuals involved in the mitigation of climate change.

A case in Germany showed that private developers of wind energy contacted some citizens in a community privately for contracts. This led to resentment among the neighbors (Jobert et al., 2007). It is possible that they felt it was unfair not to receive the same economic benefits. Resentment could also lead to changes in the decision-making process. Mizuno (2014) points to previous errors related to community engagement in wind energy development such as not taking community concerns into account. Wind farms are often placed in rural areas, thus making this conflict a fertile ground for a center – periphery conflict. Walker et al. (2018) describe a conflict in Canada where resentment is triggered through the perception that “the liberals” in cities want wind energy but none of the disadvantages. Thus, leading the opposition to conclude that the “liberals” let the people living in rural areas deal with the disadvantages.

Additionally, Q18 express an extensive censorship toward people who have spoken out against the narrative that climate change exists. This could also be a source of resentment as they feel like their rights are being oppressed. It is important to note that these are the respondents’ own perceptions and might not be an accurate representation of the situation. As we have mentioned in Section 4.4.2, the media was not interested in the facts and figures provided by the wind energy opposition. This, in combination with the media publishing facts that are perceived to be in support of wind energy, could support their idea that they are being censored. This could also lead to resentment toward people who get the media attention the opposition wants.

### 4.2 The intensity of emotional reactions and the appraisal chain

In this chapter we discuss the intensity of the emotional reactions based on the authors interpretation of the interviews. Appraisal theory (see Section 2.1) is used to discuss how these emotions arise.

We found that sadness and disgust emerged as intense emotions. For instance, respondents’ comparison of wind energy development to the Nazi invasion of Norway reflects a particularly high level of disgust. This reaction may stem from a perceived moral failing by wind energy developers, as Rozin et al. (1999) noted that contempt, anger, and disgust often are elicited by perceived breaches of moral codes. In this case, the moral breach could be tied to feelings of exploitation, as respondents perceive wind energy development as an encroachment upon nature and a loss of communal ownership of natural spaces. Frustration and anger were also prominent emotions.

Appraisal theory provides insight into how these emotional reactions occur, suggesting that emotions are often shaped by appraisal factors such as suddenness or control (Moors, 2017). For example, one respondent express surprise at the rapid onset of wind energy development in their local area, while another highlighted the lack of control – both key appraisal criteria proposed by Scherer (1999). Understanding these emotional reactions to wind energy is essential for minimizing negative impacts that could be avoided through proper care and planning.

We envision the appraisal chain to progress as shown in Figure 1. The event in question is a wind energy project, then the valence of the event is considered based on criteria ending in a positive or negative evaluation. This leads to an appraisal of different responses, which is determined by how one can deal with the consequences of the event. It then culminates in an emotional reaction that in our sample is negative. Part of the valence appraisal also includes the compatibility with personal norms, values and standards. Our results indicate that several aspects of wind energy development conflict with respondents’ norms, values, or standards. One prominent example is the breach of values and norms related to our respondents view on nature.

There was a notable difference in how freely stakeholders expressed themselves during the interviews. In general, members of the opposition were less restrained in expressing their emotions and appeared significantly more emotionally engaged than the developers. This could stem from their perception of opposing official authorities and existing decisions. Additionally, their role as neighbors of the planned or existing wind turbines likely influences their investment in the controversy. In contrast, the developers tended to be more restrained in their expression of emotions, possibly because they represent not only themselves but are interviewed in their professional capacity, or a combination of both. Even though we do not have enough participants to make broad generalizations, it was apparent during the interviews that developers often spoke on behalf of their companies, resulting in a more cautious tone, even when encouraged to speak their mind.

### 4.3 Different forms of disruption

The wide variety of emotional triggers and reactions, in addition to the opponents reasoning, supports the idea that opposition to wind energy is a multifaceted phenomenon. It is something that goes beyond the concept of “not in my backyard” (Thornton and Tizard, 2010). The developers also show triggered emotions, but their emotions are in general not as strongly felt as those of the opponents. This is to be expected as the developers are representing a business and could thus be further removed from the controversy. However, we found that many of the triggers for developers were tied to the process and to how they as part of the developers’ side are perceived.

#### 4.3.1 Disrupting access and interaction with nature

For opponents, wind turbines carry an inherent sense of disruptiveness, interpreted as a signal of human encroachment on nature. One participant expressed a belief that nature is a resource in its own right, reflecting a biospheric value orientation that values nature not for its extractable resources, but for the concept of nature and the personal connection one builds by spending time in it (de Groot and Steg, 2007). This respondent talked about the “finance-guy” view on nature, where nature is something to be used and quantified in monetary values. There was a certain resentment and anger toward this view of nature.

A possible consequence of wind energy development is loss of recreational area or a change in scenery. The change in scenery is something mentioned by our participants where they talk about “*blowing up mountains and destroying nature*” (Res 8). Referencing Figure 1, such a drastic change could be a consequence that the respondent cannot deal with leading to a negative emotional appraisal.

The change in nature is clashing with the opponent’s goal of conserving untouched nature – a theme frequently observed among our respondents (biospheric value orientation). This raises an important question for future research: How can wind power or other types of Renewable energy technology become less disruptive or is it even possible to reduce their disruptive impact on natural landscapes?

Using nature and spending time in nature has a large place in the Norwegian society. As mentioned, wind energy can disrupt the use of nature and change places in nature. This suggests that wind energy can disrupt this attachment. Our findings are in line with the suggestion of Devine-Wright and Howes (2010) who claimed that wind energy has the potential to disrupt place attachment. However, our interview data has also provided additional insight into how wind energy development may disrupt place attachment. We believe that it is through the emotional reaction known as solastalgia, which is a result of a major change in a person’s home environment (Albrecht et al., 2007). This could also be related to status quo bias. There is a general bias to keep things as they are, i.e., upholding the status quo (Samuelson and Zeckhauser, 1988).

#### 4.3.2 Disruption of the identity of Norway: colonialism and democracy

**Figure 2:**
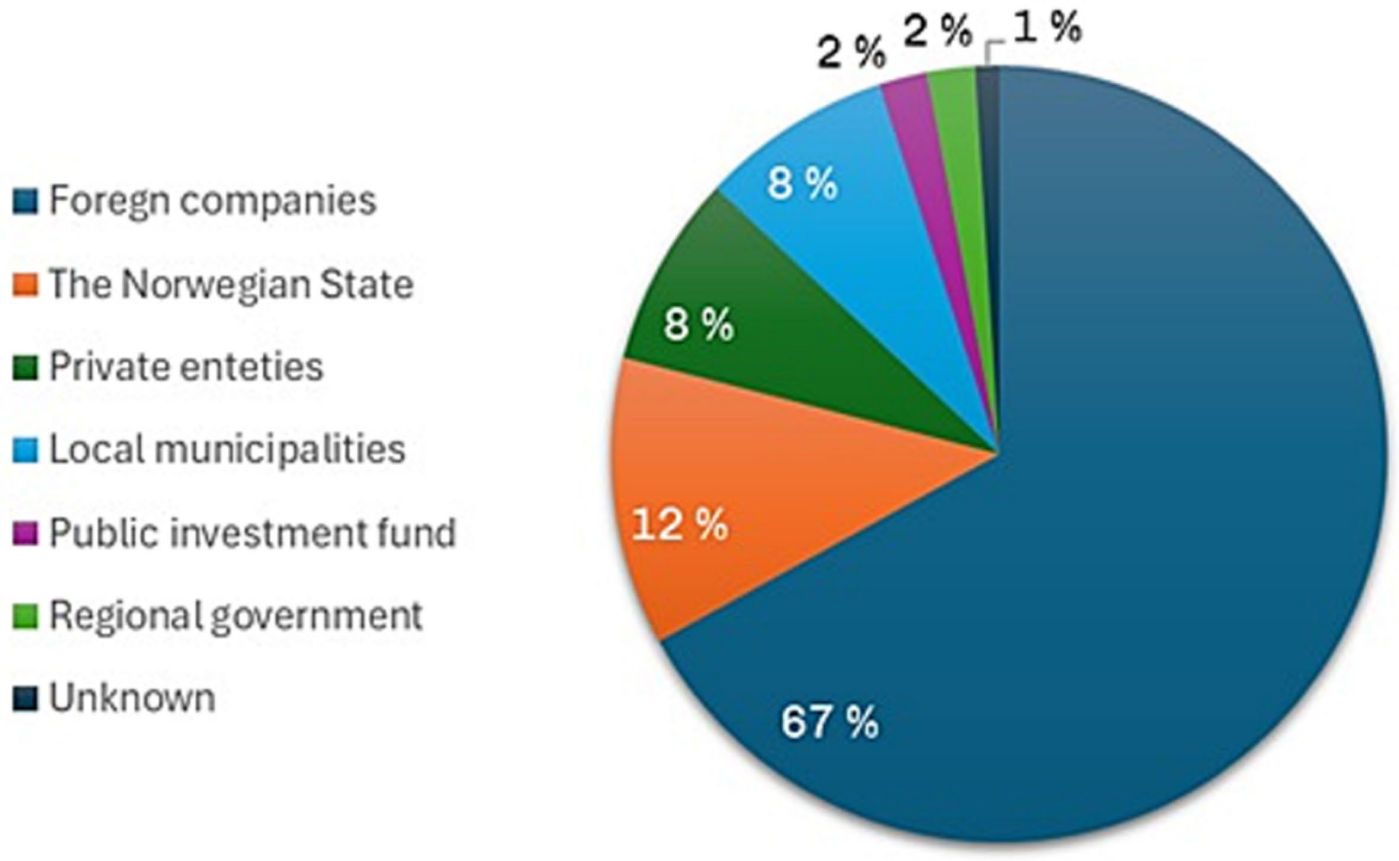
The ownership percentages of wind energy in Norway, adapted from Carlsen et al. (2023).

In many cases, investors in Norwegian wind energy projects are foreign companies, which seems to reinforce concepts of colonization and invasion. For instance, respondents 8 and 9 emphasize their concerns about foreign ownership as an appropriation of Norwegian resources, viewing wind turbines as symbol of colonization. One reason for this is the fact that 67% of Norway’s wind energy is owned by foreign investors (Pedersen, 2023), as illustrated in Figure 2. One respondent even talked about the Norway-Germany strategy and drawing parallels between establishing wind farms owned by German companies and the invasion of Norway by Nazi-Germany during World War 2. While extreme, this example shows the depth of controversy surrounding wind energy development for some Norwegians.

The idea that wind energy development is a form of colonization might seem farfetched for people not familiar with the Norwegian wind energy debate. However, Norway has traditionally had extensive control over their own natural resources (Askheim, 2024). A loss of this control could lead people to feel a lack of ownership, which may lead to a hostile attitude toward the development of wind energy. In general, a loss of control is often related to negative emotional reactions (Burger, 1989) such as helplessness which we have seen in the present study.

### 4.4 Policy implications

Our findings indicate that wind energy development can evoke strong emotional reactions, which should be carefully considered when planning, as they could impact the project’s success. While each project is unique, the sources of disruption outlined in this paper provide valuable insight into which aspects might trigger negative emotional reactions. According to Kirkegaard et al. (2023a), the inclusion of different kinds of knowledge from developers, local stakeholders and policy makers could be a key in solving the challenges that occur in the planning of wind energy developments and the further development of wind energy policy.

A common theme among developers is their disappointment with the political process, largely due to a perceived lack of political support for wind energy initiatives. While green transition is framed as a political initiative, developers feel that their own policy are not being adequately supported. This frustration with politicians is something that both developers and opponents experienced. Politicians being more engaged in the process could help lessen the frustration connected to a perceived lack of political support. Additionally, both groups face challenges in how they are portrayed in media. Developers generally report having positive relationship with traditional news outlets, but they find social media to be a much more challenging arena. The developers spoke about having to lock down their social media platforms or being extremely careful in their posts, as even unrelated content could be flooded by wind energy opponents.

Such stories might lead to a disregard, or even demonization, of opponents. Viewing engaged citizens who participate in debates about Norway’s green transition merely as obstacles to a low emission society would, from our perspective, be a significant mistake—especially if the ultimate goal is to increase the acceptance of wind energy. By recognizing engaged citizens as assets in this process, we acknowledge that controversy as an essential component of a functioning democratic system, see for instance Hess (2009). There have been instances of people creating roadblocks and setting fire to construction equipment indicating that people are very emotionally engaged and have strong convictions in their resistance toward wind farms (Thoresen et al., 2020). As discussed in the previous section on anger, these activities may be avoided if people feel included in the decision process. Politicians also need to place themselves in the wind energy debate and not shy away as we have seen in the results section a lack of involvement from politicians is also a source of frustration.

A democratic process is contingent on stakeholder engagement (citizens) and should be considered in all parts of the development process (Durham et al., 2014). The process is rather complicated involving initial proposals for regulation, decisions of the local authorities, statement on initiation from the developer, impact assessment, a preliminary decision of The Norwegian Water Resources and Energy Directorate, detailed plans from the developer and the final decision from The Norwegian Water Resources and Energy Directorate (Biong, 2023). Durham et al. (2014), further show that early stakeholder engagement could be beneficial for identifying points of conflict and conflict resolution. Our findings support the idea that engagement comes too late in the process. The late engagement cannot solely be attributed to a lack of political awareness among the local population but also reveals issues with the underlying process. Late engagement leads to less space for negotiations for people who are affected by the development. A constrained negotiation space is not conducive to a democratic process. Based on the statements from our participants, much of the frustration stems from not being heard by developers and politicians. A challenge of early engagement is who should be included in the process. Kirkegaard et al. (2023b) highlight the negative consequences of prioritizing engagement exclusively with private landowners who own the most suitable land for wind energy development. This approach often overlooks local communities, undermining the participatory nature of the development process and resulting in a less democratic outcome.

A key aspect of democracy is free discussion (Griffith et al., 1956). Increasing the negotiation space would allow stakeholders to engage with the project in a more meaningful manner, and allowing more voices to be heard earlier in the process could make it more democratic as well as feeling more democratic for those involved. This could probably limit some of the negative emotional reactions related to not feeling heard.

Changes in the ownership policy might also help address feelings of colonization and invasion associated with wind energy projects. The Norwegian population has high trust in government and skepticism toward foreign influences in business ownership (Lie, 2016). Emphasizing ownership opportunities for local inhabitants and governments in wind energy projects may be a key factor in reducing opposition. However, it is unrealistic to satisfy everyone, so some level of resistance will likely persist.

**Figure 3:**
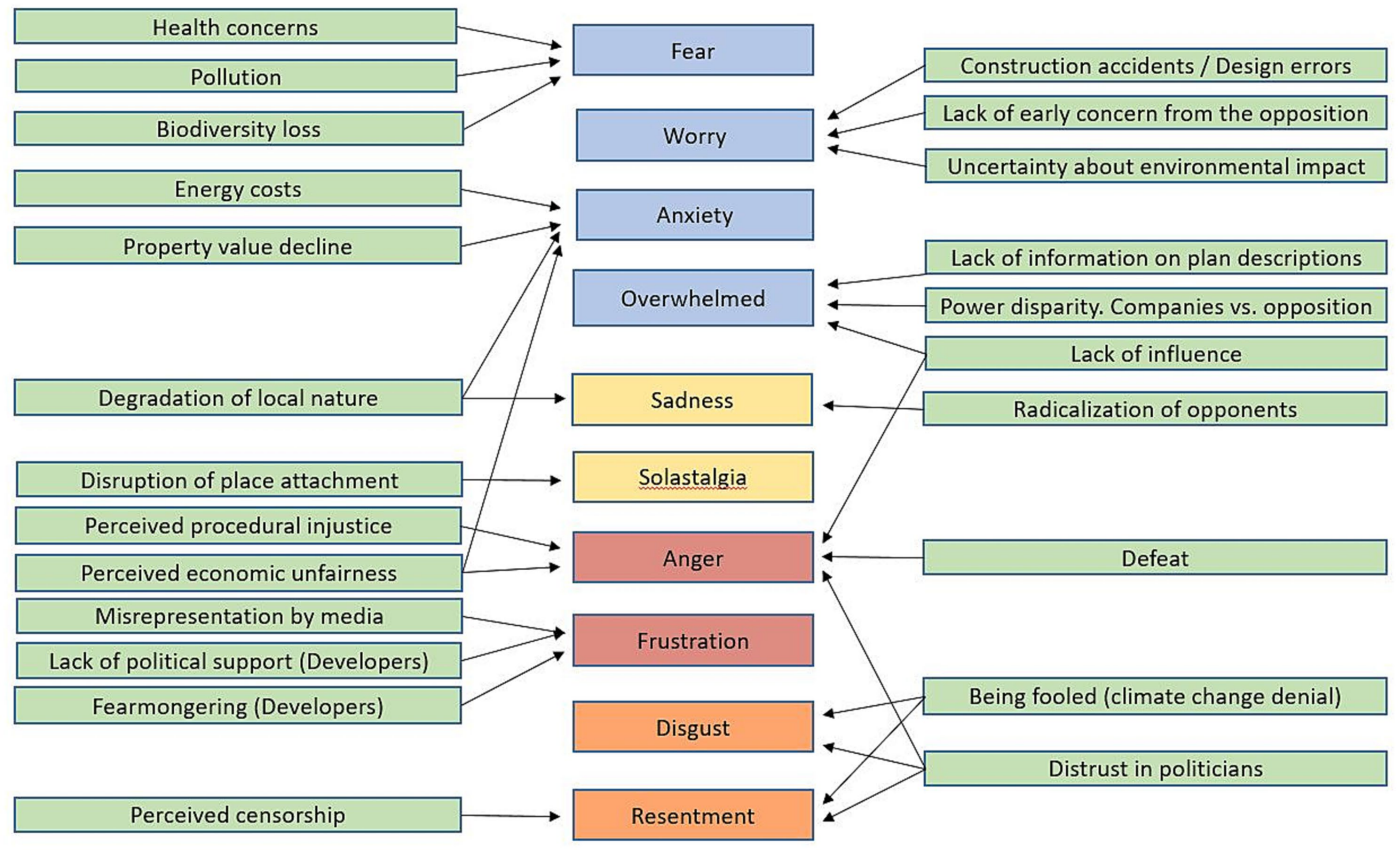
Emotions identified in this study and their associated triggers. Green represents the triggers. The emotions are labeled as follows: threat-related emotions are blue, sadness-related emotions are yellow, anger-related emotions are red, and disgust-related emotions are orange.

To summarize, this study highlights the strong emotional reactions that wind energy can provoke. These reactions, should be considered in the planning process to increase the likelihood of project success and minimize negative emotional reactions. The emotional reactions identified in this paper can be seen in Figure 3. Engaging citizens early on and viewing them as assets rather than obstacles can help reduce opposition and create a more democratic process. The study also emphasizes the importance of including all stakeholders and suggests that changes to ownership policies, such as prioritizing local involvement, could address concerns and decrease resistance.

## 5 Concluding remarks

To summarize, a total of ten different emotions and 23 triggers of these emotions were identified in the present study. The relationship between the triggers and the emotions are shown in Figure 3.

The emotions are classified into four different categories: threat-related, sadness-related, anger-related and disgust-related emotions. These categories also contain subcategories which are detailed in Table 1. Additionally, we identify several disruptive aspects of wind energy which potentially trigger emotional reactions. Wind energy development can disrupt access and interaction with nature, place attachment, the national identity of Norway and a person’s experience as a citizen and all that entails.

We emphasize the importance of stakeholder engagement throughout the development process. Early engagement is likely to reduce some of the negative emotional reactions experienced by our participants. Appraisal theory offers a valuable framework for understanding emotional reactions to wind energy projects as it sheds light on how emotions are triggered through the appraisal of external events by looking at the different appraisal factors such as suddenness and lack of control. It is important to note that these emotional reactions are probably not the results of NIMBYism or similar phenomenon (Devine-Wright, 2005) but are instead common reactions to perceived grievances.

This paper can serve as a foundation for more in-depth studies, particularly those exploring the emotional perspective of developers involved in wind energy development.

## Data Availability

The datasets presented in this article are not readily available because the raw data used in this paper is based on video and audio data. This data is impossible to sufficiently anonymize. However, transcripts can be made available upon request. Requests to access the datasets should be directed to Sigurd Hilmo Lundheim, Sigurd.h.lundheim@ntnu.com.
