# Supplementary material for: Wind energy development in Norway: exploring the emotional landscape
Source: Front Psychol. 2025 Feb 14;16:1386921. doi: 10.3389/fpsyg.2025.1386921 (PMC11868088; doi:10.3389/fpsyg.2025.1386921)
Supplement: Supplementary file 1 [file Supplementary_file_1.pdf]

Appendix 1 Interview Guide (In Norwegian)

The following is the interview guide used in the paper *Wind Energy Development in Norway: Exploring the Emotional Landscape.* The guide is presented in Norwegian, as the interviews were conducted in this language.

Spørsmål til motstandere

1. Hvordan ble du først klar over vindkraftverket?
2. Hva var dine umiddelbare tanker rundt prosjektet?
3. Hva vil du si er hovedårsaken(e) til at du stiller deg negativ til dette vindkraftverket/vindkraftparken?
4. Er du generelt negativ til vindkraft, eller finnes det vindkraft du er/kunne stilt deg positiv til?

Hva tenker du for eksempel om:

5. Plasseringen til vindkraftanlegget?
    a. Hvordan tror du de kom frem til avgjørelsen å bygge vindkraftverket der?
    b. Synes du at lokalbefolkningen har hatt en meningsfull rolle i beslutningsprosessen?
    c. Hvor mye skal lokalbefolkningen få bestemme i beslutningsprosessen?
6. Eierskap: Hvem eier vindkraftparken?
    a. Har man mulighet til å bli deleier i vindkraftparken?
        i. Har du noen tanker rundt det å bli deleier i en vindkraftpark?
            1. Enten deg selv eller andre
7. Samfunnsgoder
    a. Får lokalsamfunnet noen goder fra vindparken?
        i. Synes du at det bør være det?
            1. og hvordan ser de ut/hvordan kunne disse godene se ut?
8. Økonomiske innvirkninger
    a. Tror du vindkraftutbyggingen vil ha en økonomisk innvirkning på de lokale innbyggerne?
        i. Vil det ha en positiv eller negativ påvirkning?
9. Miljøpåvirkning
    a. Tror du at miljøpåvirkningene vil være større eller mindre enn det som er forespeilet av utviklerne?
    b. Hvordan tror du de lokale innbyggerne opplever miljøpåvirkningene?
10. Utsikten og naturbildet
    a. Tror du utsikt og naturbildet vil bli mer eller mindre påvirket enn det utviklerne forespeiler (planskisser og modeller)?
    b. Hvordan oppleves denne påvirkningen av de som bor i nærområdet?
11. Kontrovers
12. Hvordan har du opplevd motstanden?

    a. Hvordan opplever du at de rundt deg nærmeste/lokalsamfunn er innstilt til motstanden?
        i. Er det varierende ståsted, lokale diskusjoner mellom ulike beboere? Blir alle ulike reaksjoner reflektert i debatten og rapporteringen av debatten? Hvis det er ubalanse i fremstillingen, hva beror det på tror du?
    b. Hva tror du er hovedgrunnen til kontroversen?
        i. Hvilken rolle har forskjellige medier spilt i denne konflikten?
            1. Har sosiale medier spilt en rolle?
                a. På hvilken måte har sosiale medier vært viktig for mobilisering av motstanden?
        ii. Hvor viktig har mediene vært for deg når det kommer til motstand av vindkraft?
            1. Hva tror du rollen til journalister har vært i denne konflikten?
                a. Har journalistene tatt et ståsted eller har de vært nøytrale?
            2. Hvilken rolle har politikerne stilt?
        iii. Er du aktiv på sosiale medier når det kommer til vindkraft motstand?

13. Har dine holdninger til vindkraft forandret seg etter som utviklingen har pågått?
    a. Hva kunne ha blitt gjort annerledes fra motstandernes sine sider, og hva kunne ha blitt gjort annerledes fra utviklerne sine sider? Media?
14. Er det noe jeg burde ha spurt om, men som ikke kommet frem i samtalen vår?

Spørsmål til utviklere

1. Hvordan endte du opp med å jobbe med vindenergi?
    a. Har du en personlig interesse i fornybarenergi?
2. Så nå skal vi snakke litt om plasserings prosessen
    a. Hvordan går dere frem for å bestemme hvor et vindkraftanlegg skal være?
3. Videre på beslutningsprosessen
    a. Kan du gå igjennom stegene i prosessen som resulterer i avgjørelsen å bygge et vindkraftanlegg?
    b. Hvordan opplever du denne prosessen?
        i. Var det noen store hinder i denne prosessen?
    c. Vil du si at lokalbefolkning har mulighet til å være en aktiv deltaker i denne prosessen?
        i. Bør de få en mer aktiv rolle?
        ii. Hvordan?
    d. Hva tenker du om rollen til politikerne i denne prosessen, hvordan påvirker de den?
        i. Hvordan relaterer politikerne til motstandsgruppene?
4. Hvilken form for eierskap er det for de forskjellige vindparkene
    a. Er det profittdeleing eller lignende?
5. Hvilke goder får lokalsamfunnet av at dere som utvikler konstruerer en slik park?
    a. Er det noen goder du tror kunde ha gjort folk mer positive?

    b. Er der noen eksisterende/kommende goder for lokalsamfunnet som ikke er blitt kommunisert godt nok?
6. Hvilke interne følelser eller holdninger er det til utvikling av vindenergi?
    a. Har dere en bakenforliggende strategi?
    b. Hvilke restriksjoner har dere opplevd?
        i. Har det vært ressurs problemer
        ii. Personlige agendaer?
        iii. Er det noe du har hatt lyst til å gjøre i et vindkraftprosjekt som dere/du ikke har blitt hindret i å gjøre?
    c. Hvor enig er dere innad i bedriften?
        i. Finnes det noen som er mot vindkraft men fremdeles jobber med det?
7. Kontrovers
    a. Hvilken rolle har motstandsgruppene i dine øyne?
        i. Hva er grunnen til at de oppstår?
    b. Forventet dere motstand mot prosjektene?
        i. Var den sterkere eller svakere enn antatt?
    c. Hvordan opplevde du motstanden?
        i. Følte du deg personlig angrepet?
    d. Hva er den vanligste reaksjonen folk flest har?
        i. Befolkningen generelt
        ii. De i kommunene der vindparken er planlagt
        iii. Bekjente
        iv. Venner og familie
    e. Hva tror du er hovedkilden til kontroversen?
        i. Hvilken rolle har tradisjonelle medier hatt?
        ii. Hvilken rolle har sosiale medier hatt?
            1. Hvilken mediatype har hatt størst påvirkning på folk når det kommer til motstand?
                a. Hvordan er medier knyttet til motstandsgruppene?
            2. Hvordan er journalistenes rolle i denne kontroversen?
                a. Vil du si at det eksisterer bias i måten journalistene har omtalt vindkraft saker på?
        iii. Har din gruppe vært aktiv i sosiale eller tradisjonelle medier, i så fall på hvilken måte?
    f. Kunne noe ha vært gjort annerledes for å unngå kontroversen?
        i. Politikere?
        ii. Journalister?
        iii. Dere?
8. Er det noe jeg ikke har spurt om? Hva?
